# Current Concepts in Hepatocellular Carcinoma and Liver Transplantation: A Review and 2014 Update

**DOI:** 10.5005/jp-journals-10018-1123

**Published:** 2015-01-06

**Authors:** Andrew Ofosu, Ahmet Gurakar

**Affiliations:** 1 Department of Medicine, MedStar Harbor Hospital, Maryland, USA; 2 Department of Gastroenterology and Hepatology, Johns Hopkins School of Medicine, Maryland, USA

**Keywords:** Hepatocellular carcinoma, Liver transplantation, Milan criteria, University of California San Francisco criteria, Living donor liver transplantation.

## Abstract

Hepatocellular carcinoma (HCC) is the fifth most common cancer worldwide. Multiple treatment modalities for HCC are available, depending on size and number of tumor. Liver transplantation offers the most reasonable option for curative treatment, because it simultaneously removes the burden of the diseased liver. The Milan criteria currently remain the benchmark for the selection of patients with HCC for transplantation. However, there is considerable and promising interest in expanding the eligibility criteria to include the University of California San Francisco criteria. Liver transplantation (LT) has progressed during the past decade. The introduction of living donor LT has provided a means of expanding organ transplant, but with some inherent concerns. Herein, we reviewed the diagnosis and management of HCC with emphasis on the current concepts of liver transplantation for the treatment of HCC.

**How to cite this article:**

Ofosu A, Gurakar A. Current Concepts in Hepatocellular Carcinoma and Liver Transplantation: A Review and 2014 Update. Euroasian J Hepato-Gastroenterol 2015;5(1):19-25.

## INTRODUCTION

Hepatocellular carcinoma (HCC) is currently the third leading cause of cancer-related deaths worldwide.^1,2^ Despite these serious statistics, improvement in patient stratification and introduction of new-targeted therapies has increased survival and transformed the treatment modalities for HCC.

Several therapies have been proposed for these patients with proven survival benefits in the early stages of HCC. These therapies comprise surgical resection, various locoregional treatments, including percutaneous ethanol injection, radiofrequency ablation (RFA), trans-arterial chemoembolization (TACE), and radioemboliza-tion.^3,4^ However, liver resection (LR), liver transplantation (LT) and percutaneous tumor ablation are currently considered as curative treatment modalities for HCC in different disease stages. Nevertheless, patients who underwent resection and ablation remain at high risk of recurrence and development of new lesions.

The first successful human orthotopic liver transplant (OLT) was performed by Dr Thomas Starzl in 1967. Liver transplantation became the standard of care for end-stage liver disease in the 1980s, particularly with the introduction of various immunosuppressants. At present, the success of OLT is marked by 1 and 5-year survival rates of 85 and 70%^5^ respectively. Furthermore, in carefully selected subgroup of patients, approximately 83 to 92% of patients are recurrence free.

## EPIDEMIOLOGY OF HCC

The pattern of HCC occurrence has a clear geographical distribution, with the highest incidence rates in East Asia and sub-Saharan Africa, where around 85% of cases occur due to the endemic high prevalence of hepatitis B and C. Low incidence areas include North and South America, most of Europe, Australia, and parts of the Middle-East, with fewer than three cases reported per 100,000 population per year. A total of 48,596 cases of HCC were reported in the United States between 2001 and 2006 according to data from the Surveillance, Epidemiology and End Results (SEER) program of the National Cancer Institute and the National Program of Cancer Registries from the centers for disease control and prevention.^6^ The incidence of HCC increases progressively with advancing age in all population, reaching a peak at 70 years.^7^ Hepatocellular carcinoma has a strong male preponderance with a male to female ratio estimated as 2:4.^8^

## RISK FACTORS FOR HCC

Any chronic liver injury can potentially increase the risk of HCC. This risk appears elevated particularly in patients who develop cirrhosis. The most frequent factors include chronic viral hepatitis (types B and C) and alcoholic liver disease. In Africa and East Asia, the largest attributable fraction is due to hepatitis B (60%), whereas in the developed western world, only 20% of cases can be attributed to HBV infection, and chronic hepatitis C appears to be the major risk factor.

Recently, risk factors associated with the metabolic syndrome have been recognized as potential causes of nonalcoholic hepatosteatosis, cirrhosis and, thus, may lead to HCC. Other less common risk factors include exposure to foods contaminated with aflatoxins or other environmental toxins that are considered hepatocarcino-genic, including nitrosamines, carbon tetrachloride, and polyvinyl chloride.

Dietary exposure to aflatoxin B1, derived from the fungi *Aspergillus flavus* and *A. parasiticus,* is an important contributing factor for HCC development in some parts of Africa and Asia. Epidemiological studies have shown a strong correlation between the dietary intake of aflatoxin B1, P53 mutations, and the incidence of HCC specifically in HBV-infected individuals.^9^

Metabolic disorders, including hemochromatosis, alpha 1-antitrypsin deficiency, type I glycogen storage disease, citrullinemia, porphyria, tyrosinemia, and Wilson disease develop into HCC most often with a background of cirrhosis.

## PROTECTIVE FACTORS

Some observational studies have shown that coffee consumption is a protective factor against HCC. This protective effect is probably because of the presence of antioxidants and the effects of lowering the aminotransferase levels.^10^ Higher dietary intake of vitamin E has also been associated with a decreased risk of liver cancer among patients both with and without a self-reported history of liver disease or a family history of liver cancer.^11^

## DIAGNOSIS OF HCC

The approach to the diagnosis of HCC has been outlined in a recent consensus statement issued by the American Association for the Study of Liver Disease (Flow Chart 1). The European Society for Gastrointestinal Endoscopy recently released similar guidelines, which differ in the evaluation of patients with nodules between 1 and 2 cm in size.^12^ The current approach for diagnosis is based largely on noninvasive testing.

**Flow Chart 1:**
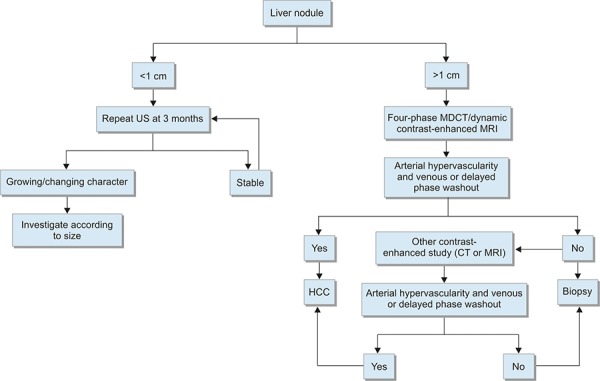
Diagnostic algorithm for suspected HCC (CT: Computed tomography; MDCT: Multidetector computed tomography; MRI: Magnetic resonance imaging; US: Ultrasound). Used with permission from AASLD^3^

### Noninvasive Testing

The diagnosis of HCC can be made using noninvasive imaging tests. A definitive diagnosis can be established based on the presence of typical imaging features showing areas of early arterial enhancement and delayed washout (less enhancement than the rest of the liver) in the venous or delayed phase of four-phase multidetec-tor computed tomography (MDCT) (the four phases are unenhanced, arterial, venous and delayed) or in dynamic contrast-enhanced magnetic resonance imaging (MRI).

These radiological changes are related to increased vascularity within the HCC lesion, which is supplied by the hepatic artery. For lesions between 1 and 2 cm in diameter, concordant findings from CT and MRI are recommended to establish the definitive diagnoses of HCC.

### Pathological Diagnosis

Percutaneous biopsy should only be performed when diagnostic imaging results are uncertain, e.g. in patients with cirrhosis with iso- or hypovascular enhancing lesions during the arterial phase without washout. Further, the result would have a direct impact on management.^13,14^ Major complications of biopsy, such as bleeding and needle tract seedlings, have been reported in some cases.

### Molecular Markers in Diagnosis

Although elevated serum alpha-fetoprotein (AFP) can also be observed in patients with chronic liver disease without HCC, such as acute or chronic viral hepatitis, an increase in serum AFP in a patient with cirrhosis should raise the suspicion of HCC. It is generally accepted that serum levels greater than 500 mcg/l in a high-risk patient is diagnostic of HCC.^15^ However, not all tumors secrete AFP, and serum concentrations are normal in up to 40% of small HCCs. Alpha-fetoprotein levels are normal in the majority of patients with fibrolamellar carcinoma, a variant of HCC.^16^

Despite the concerns regarding the use of AFP as diagnostic marker for HCC, it serves as an important preoperative prognostic marker, particularly in patients undergoing resection as well as those being considered for LT. Patients with AFP levels >1000 mcg/l are at an extremely high risk of recurrent disease following transplantation, irrespective of the tumor size.^17,18^

Additional serum markers currently being explored include tumor-associated isoenzymes of gamma-glutamyl transpeptidase, urinary transforming growth factor-beta 1, and serum levels of circulating intercellular adhesion molecule-1. These serum markers are currently under investigation, but have been included as part of the current guidelines for diagnosis.

Other markers, such as plasma microRNA expression, are also under study as possible markers for HCC.^19^

## STAGING AND PROGNOSIS OF HCC

The most commonly used systems are the TNM, Okuda and Barcelona systems, and the Cancer of the Liver Italian Program score (CLIP score).^20-22^ These four systems for staging invariably include the severity of underlying liver disease, tumor size, tumor extension into adjacent structures and the presence of metastases. Once HCC is diagnosed, staging with either CT or MRI of the chest, abdomen and pelvis is required.

The Barcelona Clinic Liver Cancer (BCLC) system includes performance status, presence of multifocal tumor lesions, vascular invasion, extrahepatic spread, Child-Pugh stage, and portal hypertension.^23^ Recent studies have deemed this as the best prognostic system; however, it is limited with respect to not being patient-centered, but being algorithmic.^24,25^

Currently, there is no consensus as to which staging system is best in predicting the survival of patients with HCC. The Americas Hepato-Pancreato-Biliary Association recommends the use of the TNM system to predict outcome following resection or LT, and the BCLC scheme for patients with advanced HCC who are not candidates for surgery.^26^

Other survival determining factors include whether patients are living in high-incidence *vs* low-incidence areas, the histological grade of differentiation and serum AFP levels at diagnosis.

## TREATMENT OF HCC

### Liver Transplantion

The only potential curative treatment modalities of HCC currently are LT and LR. However, patients who underwent a resection remain at risk of recurrence and development of new lesions. Other treatment options also present a higher long-term risk of recurrence because they have no effect on chronic liver disease, which is the major driving factor in the development of HCC.

Dr Thomas Starzl performed the first liver transplant in humans in 1963. Early experiences with LT for HCC were dismal with high recurrence rates and high 90-day mortality rates, reflecting the fact that the selected patients had advanced disease.^27,28^

The second period of development started in the early 90s, when data reassessment suggested that patients with incidental and asymptomatic HCC may achieve outcome comparable to patients with nonmalignant liver disease. Although randomized trials have not been conducted, uncontrolled series suggest that survival following LT is as good or better than after alternative treatments for HCC in carefully selected patients.^29-31^ Further, a survival benefit was observed in patients undergoing OLT as compared to that of other treatments: 3 and 5-year survivals were 72 and 68% for OLT, as compared to 64 and 44% for resection, 54 and 36% for percutaneous ethanol injection, and 32 and 22% for TACE respectively.^29^

### Eligibility Criteria: Milan and Beyond

The landmark study of Mazzaferro in 1996 was a significant advancement for LT, by which deceased donor liver transplantation (DDLT) was established as a viable option for the treatment of HCC.^32^ This led to the development of the Milan criteria, with LT restricted to patients with early HCC (defined as single lesion <5 cm, up to three separate lesions, none larger than 3 cm, no evidence of gross vascular invasion, and no regional nodal or distant metastases).

Using explant pathological data, Yao et al and researchers at UCSF demonstrated that patients with a single lesion less than 6.5 cm, or up to three lesions each less than 4 cm, with a cumulative diameter less than 8 cm and having surgical outcomes similar to those transplanted under the Milan criteria.^33^ However, its implementation has been associated to some criticism. The University of California San Francisco criteria were based on retrospective data analysis. Some patient characteristics were very heterogeneous because several of them underwent pretransplant TACE for tumor downstaging, whereas others did not, and data were based on histo-pathology analysis, which does not correspond with the clinical decision process.

In a retrospective multicenter trial, the Milan group recently reported the outcome of 1,112 liver transplant patients with HCC exceeding the Milan criteria on histo-pathology examinations compared with 454 matched patients with HCC (the Metroticket project).^34^ Five-year overall survival for those patients exceeding the criteria was 53.6% compared to 73.3% for those that met the criteria. The authors identified a subgroup of 283 patients without microvascular invasion, who met the new created so-called ‘up-to-seven’ criteria [HCC with 7 as the sum of maximum size of the largest tumor (in cm) and the number of tumors] that achieved an excellent 5-year survival of 71.2%, in accordance with the results applying Milan criteria.^34^ Further data showed a linear effect of hazard ratios with tumor size.

This clearly illustrates that the expansion of the selection criteria beyond the Milan criteria is limited by an increase in recurrence and decrease in survival.

Recent collected data from 156 liver transplants performed at Johns Hopkins Medical Center, between August 2000 and August 2013, showed that Milan and AFP were less predictive of explant microvascular invasion. Multi-variable logistic regression demonstrated that patients with multilobar lesions had four times greater odds of microvascular involvement even after controlling for Milan criteria and AFP [odds ratio (OR) 4.17, p = 0.047].^35^

### Allocation of Donor Organs

Due to the limited number of donors, an allocation scheme has been developed to streamline the allocation of donor organs to the most severely ill patients. In the United States, allocation of deceased donor organs is based upon the model for end-stage liver disease (MELD) score, a statistical model based upon predicted survival in patients with cirrhosis. Given its accuracy in predicting short-term survival among patients with cirrhosis, MELD was adopted by the United Network for Organ Sharing (UNOS) in 2002 for prioritization of patients awaiting LT in the United States. A high MELD score indicates a high short-term mortality. In patients with HCC, the use of the traditional MELD criteria is limited because in patients with HCC, prolonged waiting often results in tumor growth as well as progression of the underlying liver disease, which may lead to disqualification from the transplant list.

Under the current UNOS policy for allocation of deceased donor livers for transplantation, patients with HCC (single HCC between 2 and 5 cm, or two to three lesions, none greater than 3 cm) who are potential OLT candidates are assigned a MELD score of 22, indication of a higher mortality rate, leading to priority access to a donor liver. Patients not receiving a transplant in the first 3 months of listing with a MELD score of 22 are upgraded to a score of 25 as long as they continue to meet the established national criteria for HCC.

### Management of HCC while on the Waiting List

The limited availability of donor organs has led to the widespread use of locoregional therapy for HCC while patients await transplantation.

The common types of treatment include RFA and TACE. Both types result in the ablation of tumor tissue through heat (RFA) or local ischemic necrosis (TACE). Transarterial chemoembolization involves the injection of chemotherapeutic agents often in combination with lipiodol into the hepatic artery branch feeding the tumor. TACE has been employed in the treatment of HCC for many years and several earlier studies have shown that TACE can help to prevent dropout from the LT waitlist because of tumor progression.

There are several single-center reports indicating that these techniques may be advantageous in preventing tumor progression beyond the Milan criteria, which often results in the loss of HCC exception points and priority, and sometimes even removal from the waiting list.^36-40^ Although there are less data for TACE, radioembolization using yttrium-90-labeled microspheres (Sirtex Medical, Sydney, Australia) has been shown to limit disease progression, which may allow patients more time to wait for a donor organ.

Periodic waiting list monitoring should be performed by imaging (dynamic CT, dynamic MRI or contrast-enhanced ultrasonography) and AFP measurements. Patients with progressive disease in whom locoregional intervention is not considered appropriate or is ineffective should be removed from the waiting list.

### Role of Bridging Therapy

The goal of downstaging using locoregional therapy, e.g. alcohol injection, RFA, TACE, transarterial radioemboli-zation or LR, is to decrease the tumor size and number in patients initially presenting with tumors that do not meet locally acceptable criteria for LT.

Some prospective studies showed that survival after LT in patients with large tumor burden successfully treated by downstaging was similar to survival in patients who initially met the criteria for transplantation.^39^ Thus, LT may be considered after successful downstaging, and it should achieve a 5-year survival comparable to that of patients with HCC who met the criteria for LT without downstaging treatment.

### Post-transplant Management

The main concern after a successful LT is the recurrence of tumor. When recurrence occurs, it is usually observed within the first 2 years after LT. Guidelines from the National Comprehensive Cancer Network (NCCN) suggest the following after LT:

- Computed tomography or MRI every 3 to 6 months for 2 years, then annually
- Serum AFP assay, if initially elevated, every 3 months for 2 years then every 6 months.

A meta-analysis of various studies showed that, compared to a sirolimus-free regimen, the use of a sirolimus-based regimen significantly decreased overall tumor recurrence (OR 0.30, 95% CI 0.16-0.55) and significantly lowered recurrence-related mortality (OR 0.29, 95% CI 0.12-0.70).^41^ Sorafenib, a multitargeted tyrosine-kinase inhibitor, was shown to exert an antitumor effect in patients with advanced HCC.^42^ Currently, the STORM trial is ongoing to evaluate efficacy and safety of sorafenib *vs* placebo as adjuvant treatment of HCC after potentially curative treatment (surgical resection or local ablation).

Hepatocellular carcinoma recurrence after LT may be treated by surgery for resectable lesions or by locoregional therapy or systemic therapy (including sorafenib) for unresectable lesions. Liver retransplantation is not considered an appropriate treatment for recurrent HCC.

### Living Donor Liver Transplant

The limiting factor for LT is availability of deceased-donor organs. The introduction of the Living donor liver transplant (LDLT) has provided a means of expanding organ transplantation. The advantages of LDLT included reduction of cold and warm ischemic times, and significant reduction of pretransplant waiting times.

A single-center study that compared 153 LDLT recipients and 350 deceased-donor recipients, who had similar MELD scores at the time of listing, found that LDLT patients had shorter waiting times, lower MELD scores at the time of transplantation, and a 1 year survival advantage from the time of listing.^43^ However, some data have suggested a higher risk of tumor recurrence with the use of partial grafts from living relatives *vs* whole grafts from deceased donors.^44^ Six studies compared DDLT and LDLT for HCC, and a higher risk of recurrence was noted in fast-tracked patients possibly because the short interval between diagnosis and LT may not allow enough time for the biological behavior of the tumor to manifest.

Although LDLT is very promising, there are some issues that have to be addressed. These include risk of hepatectomy related morbidity and mortality and ethical and psychosocial considerations regarding both donor and recipient. LDLT must be restricted to centers of excellence in liver surgery and LT to minimize donor risk and maximize recipient outcome. LDLT is acceptable for patients with HCC who have an expected 5-year survival similar to comparably staged patients receiving a deceased donor liver.

In patients following LDLT for HCC within the accepted regional criteria for DDLT, retransplantation for graft failure is justified; however, in patients beyond accepted regional criteria for DDLT, retransplantation for graft failure using a deceased-donor organ is not recommended.

### Eligibility Criteria for LDLT Currently used at Johns Hopkins Liver Transplant Program

Candidates medically eligible for DDLT can be considered for LDLT if they qualify for the criteria outlined below:

- Patients with HCC diagnosed by imaging according to the Milan criteria with age ≤60 years and biological MELD ≤22. Bridging therapy may or may not be required.
- Patients beyond the Milan criteria, who have undergone downstaging should be ≤60 years, have a MELD ≤22, with no extrahepatic disease or vascular invasion, AFP ≤400 or have well-differentiated lesion on biopsy. Bridging therapy may or may not be required.
- Patients with a T1 lesion (solitary lesion less than 2 cm on imaging without vascular invasion) with age ≤60 years and MELD 22. Bridging therapy is not required.

## CONCLUSION

Liver transplantation offers an effective treatment strategy for HCC. Careful selection of patients based on the LT eligibility criteria is critical and optimization of pretransplant therapies is important to minimize post-transplant recurrence and maximize long-term survival. Expansion of eligibility beyond the Milan criteria, using the UCSF criteria and ‘up-to-seven criteria’ seems promising.

Although expanding the criteria for OLT allows eligibility of a larger number of patients for LT, arguments against expanding the criteria include increased risk of vascular invasion and tumor recurrence at higher stages of HCC. Patients with advanced HCC exceeding the UCSF/Milan criteria can be downstaged to fit the criteria using locoregional therapy. Multicenter clinical trials should be focused on morphological, pathological, and biological parameters to minimize failure of LT. The further development of LDLT has allowed more patients to benefit from OLT with reduced waiting time on the transplant lists with favorable results.
